# First Complete Genome Sequence of a Distinct Papaya Ringspot Virus Isolate from the Northeastern Region of India

**DOI:** 10.1128/genomeA.00437-18

**Published:** 2018-05-31

**Authors:** Ritesh Mishra, Basavaprabhu L. Patil

**Affiliations:** a ICAR-National Research Centre on Plant Biotechnology, IARI, New Delhi, India

## Abstract

This is the first report of a Papaya ringspot virus (PRSV) isolate from the northeastern region of India. The nucleotide sequence identity of PRSV-Meghalaya was in the range of 72.6 to 82.5% with other Indian PRSV isolates, and the highest identity of 84.4% was with a French isolate. Population genetic analysis indicated positive selection.

## GENOME ANNOUNCEMENT

Papaya ringspot virus (PRSV) belongs to the family Potyviridae and the genus Potyvirus, with a positive-sense single-stranded RNA genome of ∼10,349 nucleotides (nt) in size, encompassing a single open reading frame (ORF) (1–5). PRSV is classified as either PRSV-P or PRSV-W type, depending on whether it infects papaya or cucurbits, respectively (6–8). PRSV is hypothesized to have originated in India ∼2,250 years ago, spread to East Asia ∼600 years ago, and reached the Americas ∼200 years ago (9). Although ∼5 complete PRSV sequences have been published from India (10, 11), none of them are from the northeastern region (NER) of India.

In 2015, leaf samples from a PRSV-infected papaya plant were collected from Umiam (erstwhile Barapani) in the state of Meghalaya, located in the NER of India. Total RNA extraction and cDNA synthesis were done as described by Patil et al. (12). Later, the cDNA was used for PCR amplification with potyvirus universal primers (13) and degenerate primers from this study. Cloning and sequencing of the coat protein (CP) and helper component proteinase (HcPro) regions indicated it to be a distinct sequence. Later, degenerate primers were designed at conserved regions of PRSV, and the entire PRSV-Meghalaya genome was real-time PCR (RT-PCR) amplified as 5 segments of ∼1.8 to 2.5 kb in size. These amplicons were cloned and sequenced as described by Patil et al. (12). The assembly of the contigs resulted in a complete genome size of 10,343 nt for the PRSV-Meghalaya isolate.

PRSV-Meghalaya was subjected to phylogenetic analysis, along with 21 full-length sequences of PRSV isolates from all over the world (12, 14, 15). PRSV-Meghalaya clustered with isolates from France, Mexico, Brazil, and the United States, in addition to all the other Indian PRSV isolates. It showed the highest nucleotide sequence identity of 84.4% with PRSV-France-E2, while the lowest sequence identity (70.6%) was with PRSV-South Korea (16), whereas the corresponding amino acid sequence identities were 89.5% with PRSV-France-E2 and 86.7% with PRSV-South Korea. PRSV-Meghalaya showed 82.5%, 81.7%, 77%, and 72.6% homology with nucleotide sequences of the four Indian isolates of PRSV (10, 11), namely, Pune, Delhi, Hyderabad, and Rajasthan, respectively.

To identify the most variable region of PRSV-Meghalaya, SimPlot was drawn for the sequences of 21 PRSV isolates (17). The genomic region of nt 241 to 1821 corresponding to P1 and nt 8841 to 9501 corresponding to the overlapping region of NIb-CP were the most variable. Recombination breakpoints were identified at 3 sites of PRSV-Meghalaya, namely nt 905 to 1134, nt 8844 to 9996, and nt 9205 to 10182, when analyzed using the RDP4 program (18). The degree of selective constraints at the amino acid level was estimated with MEGA 5.05 by analyzing separately the rate of nonsynonymous (*dN*) and synonymous (*dS*) substitutions with the Pamilo-Bianchi-Li method (19), and DnaSP 5.10 was used to evaluate the importance of natural selection (20). The proportion of nucleotide substitution between the nonsynonymous and synonymous sites (*dN*/*ds*) was 1.8, indicating a positive selection for the amino acid sequence conservation in PRSV-Meghalaya (21). This is the first report of a full-length sequence of a PRSV isolate from the NER of India, indicating its independent evolution.

### Accession number(s).

The complete genome sequence of PRSV isolate Meghalaya has been deposited in NCBI GenBank with the accession number MF356497.
